# Early Regulatory Skills and Social Communication Development in Infants with Down Syndrome

**DOI:** 10.3390/brainsci11020208

**Published:** 2021-02-09

**Authors:** Emily K. Schworer, Deborah J. Fidler, Lisa A. Daunhauer

**Affiliations:** 1 Division of Developmental and Behavioral Pediatrics, Cincinnati Children’s Hospital Medical Center, Cincinnati, OH 45229, USA; 2 Human Development and Family Studies, Colorado State University, Fort Collins, CO 80523, USA; deborah.fidler@colostate.edu (D.J.F.); lisa.daunhauer@colostate.edu (L.A.D.)

**Keywords:** Down syndrome, regulatory function, social communication, infants

## Abstract

Children with Down syndrome (DS) demonstrate substantial variability in communication and language outcomes. One potential source of variability in this skill area may be early regulatory function. Characterizing the early link between regulatory function and early social communication may benefit infants with DS at risk of difficulties with social communication and language skill acquisition. Forty-three infants with DS were assessed at two time points, six months apart. At Time 1, the average chronological age was 9.0 months (SD = 3.9) and caregivers completed the Infant Behavior Questionnaire-Revised (IBQ-R) to assess regulatory function. Six months later, caregivers rated infant communication at the second visit using the Communication and Symbolic Behavior Scales Infant Toddler Checklist (CSBS-ITC). Infant developmental level was assessed at both visits using the Bayley Scales of Infant and Toddler Development, Third Edition and caregivers reported on developmental history and biomedical comorbidities. Infant regulatory function at Time 1 predicted social communication outcomes at Time 2, six months later. Findings from this study suggest that elevated risk for pronounced communication challenges may be detectable as early as infancy in DS.

## 1. Introduction

Down syndrome (DS) is associated with increased risk for cognitive and behavioral dysregulation [1,2,3,4]. Difficulties with the cognitive foundations of regulated, goal-directed behavior, commonly referred to as “executive function” (EF), are prevalent among children, adolescents, and adults with DS [5]. School-age children with DS demonstrate a pattern of difficulties with aspects of EF on both laboratory and ecological measures, often featuring difficulties with the memory and planning subcomponents of this cognitive skillset [3,4,6]. These difficulties impact participation in everyday contexts, as EF has been linked to adaptive behavior in academic settings among school-age children with DS [7] and employment outcomes in adults with DS [8].

Similarly, difficulties with communication and language are common among individuals with DS, with implications for participation in academic, social, and community settings [9]. While infants with DS tend to show early inclinations toward social vocalizing [10], on average, young children with DS show delays in the onset of spoken first words [11] and more pronounced challenges in expressive language development than in cognitive skill acquisition [12,13]. The social communicative foundations that facilitate language acquisition in DS are also unique in presentation. Children with DS tend to use eye contact, gesture, and vocalizing for social sharing purposes (e.g., joint attention), but less so for instrumental purposes and regulating others’ behavior (nonverbal requesting) [14]. Importantly, there is a great deal of heterogeneity in communication and language outcomes in DS, with some individuals developing phrased speech and pragmatic skills, and others who show minimal verbal skills and infrequent use of vocalization for social purposes [11,15]. Understanding the sources of heterogeneity in language and communication outcomes in DS will facilitate a richer understanding of developmental trajectories and can inform more effective treatment to improve adaptive communication in this population.

### 1.1. Self-Regulation

One hypothesized source of variability in the development of communication and language in DS is individual differences in self-regulation. Though there is an ongoing discussion in the literature regarding the development of self-regulation in childhood, it generally encapsulates the ability to control behavior and responses in adaptive ways across a variety of dimensions, including attention, emotion, behavior, and cognition [16,17]. Self-regulation is also an umbrella term that subsumes a variety of constructs at different stages of development. During infancy (3–12 months), the early origins of self-regulation are first observed in the modulation of arousal states, and then in terms of infant responses to inputs [18]. Recently, Bedford and colleagues (2019) termed this early infant response modulation as “regulatory function” [19]. The development of motor skills, attention, and emotion subsequently influence the development of regulatory capacities [16], as infants and toddlers begin to recruit these skills in the service of selecting responses like looking away, and later crawling or walking away, from aversive stimuli, and approaching favorable stimuli. As infant behavior becomes increasingly more goal-directed during early childhood, higher order cognitive control (in the form of EF) becomes a critical aspect of self-regulation [18]. EF is primarily understood as a set of higher-order cognitive processes that are necessary for goal-directed behavior. Although there is growing literature connecting early infant regulatory and cognitive foundations to EF skills [20,21], the integration of working memory, planning, inhibition, and flexibility in concert with one another is generally thought to have its onset after infancy.

#### 1.1.1. Regulatory Function

Research on typical and atypical childhood development has demonstrated an important relationship between EF and both communication [22] and language skills [23]. However, only recently have researchers begun to trace this relationship back to its earliest manifestations during infancy. Early regulatory function, as a hypothesized precursor of EF, may be influential as communication foundations emerge throughout infancy [19,24,25]. Regulatory function can be captured through infant affective, attentional, and behavioral responses to the various inputs they experience in their daily environments [18], for example, disengagement of attention when presented with an aversive stimulus. In this way, early regulatory function may influence the frequency and quality of early social engagement and the degree to which an infant sustains interactions, which in turn may impact the nature of the contexts wherein social communication skills emerge.

#### 1.1.2. Regulatory Function and Communication

In typical development [26], and more recently in clinical populations (i.e., autism spectrum disorder (ASD) and premature infants) [19,27], aspects of regulatory function have been shown to predict the development of social communication in the form of joint attention and nonverbal requests. Elevated risk for early difficulties with regulatory function may place some infants with DS at a more pronounced risk for downstream challenges in communication and language.

Existing work on self-regulation in DS suggests elevated risk for under-responsivity and lower levels of initiation during early development and throughout childhood and adolescence [28,29,30]. Young children with DS often show difficulties with the early development of planful, goal-directed actions on objects [14,31,32]. Toddlers with DS produce less parsimonious strategies on tasks that require early planning with objects than other toddlers at similar developmental levels [31], and infants with DS are more likely to produce less efficient goal-directed action plans on means-ends tasks than their typically developing counterparts [32]. In addition, concurrent correlations between the parsimony of early action planning and rate of early social communication behaviors are observed in toddlers with DS [14]. These findings suggest elevated risk for early cognitive and behavioral dysregulation in DS and their potential implications for variability in communication skill acquisition; however, they focus only on the modulation of goal-directed action planning, and much is still unknown regarding infant regulatory function more broadly in this population.

#### 1.1.3. Measuring Regulatory Function

Because early regulatory function is a dimension that encompasses a behavioral/response style across a range of situations, it has been measured by using caregiver report questionnaires that focus on infant behavior, rather than laboratory observation [24,33]. The Infant Behavior Questionnaire-Revised (IBQ-R) [34] is a commonly used measure of infant temperament that has a well-established factor structure. One of the three broad factors of the IBQ-R is Orienting/Regulation, which has recently been identified as a key measure of regulatory function [19]. The Orienting/Regulation dimension was derived from a factor analysis and includes the following IBQ-R scales: Duration of Orienting, Cuddliness/Affiliation, Low Intensity Pleasure, and Soothability. Duration of Orienting characterizes infants’ ability to sustain attention during activities such as play or engagement with objects and includes the degree to which an infant attends to adults while engaging in household activities. The Cuddliness/Affiliation dimension measures an infant’s enjoyment and positive responsivity to being held by a caregiver. Low Intensity Pleasure focuses primarily on positive infant responses to gentle and soothing activities such as being read to or listening to musical toys in their crib, and Soothability involves the degree to which an infant reduces fussiness or distress in response to caregiver behavior, such as singing or rocking. These areas cohere around an infant’s ability to organize responses to their environment and adapt their attention and behavior in modulated ways. We note here that the Orienting/Regulation dimension of the IBQ-R has been examined in many studies to date, and it is in recent work that the ‘regulatory function’ terminology has been linked to this broad factor [19].

### 1.2. Clinical Implications

Examining the link between early regulatory function and subsequent social communication may help to identify young children with DS at risk of pronounced communication delays. The examination of this association should also incorporate additional factors such as prematurity, congenital heart disease (CHD), and parent education level, each of which may impact social communication. Prematurity and CHD are common in DS [35,36] and parent education level has been connected to language outcomes in typically developing children [37].

The link between regulation and communication may also yield important clinical insights. Early dysregulation may play a yet unexamined role in the elevated risk for comorbid psychiatric conditions in DS, including ASD. Among infants at risk of ASD in the general population (infants with an older sibling with ASD), infant regulatory function predicts ASD symptomatology at 3 years and at 7 years [19,38], and regulatory function moderates the association between infant symptom presentation and subsequent ASD presentation during middle childhood [19].

Despite the extensive challenges associated with comorbid DS and ASD, detection of risk for this comorbidity has not been extensively researched. The diagnostic criteria for ASD include atypical presentation in two critical areas: (1) social communication and (2) restricted, repetitive patterns of behavior, interests, or activities. The early emergence of restricted and repetitive behavior is challenging to differentiate from normative ritualistic behavior in infants and toddlers, and even more difficult to differentiate among young children with DS who do and do not have ASD. However, social communication skills are readily observable and can be evaluated among all children during early development, and there are well-validated measures to assess these skills in typical and clinical populations. Measures of early social communication, such as the Communication and Symbolic Behavior Scales Infant Toddler Checklist (CSBS-ITC), have been successfully used in the general population to predict ASD outcomes [39]. Thus, an examination of the early associations between regulatory function and social communication in infants with DS will make an important contribution to the broader effort to understand developmental trajectories for critical EF and communication outcomes in young children with DS, and may potentially aid in the detection of risk for challenging comorbidities [40,41].

In this study, we examine the relationship between infant regulatory function and subsequent social communication during early development in DS. Specifically, we inspect the score distributions of two caregiver report measures, the Orienting/Regulation dimension of the Infant Behavior Questionnaire-Revised (IBQ-R) [34] and the Communication and Symbolic Behavior Scales Infant Toddler Checklist (CSBS-ITC) [42]. The effects of prematurity [43] and CHD [36], both biomedical risk factors associated with DS, as well as caregiver education level, are also investigated. We then characterize the predictive value of infant regulatory function for social communication performance six months later. Examining this relationship offers insight into clinical practice by defining areas of risk in regulatory function associated with early social communication skill acquisition and may help identify those infants with DS at greatest risk of more pronounced delays in communication and language acquisition.

## 2. Materials and Methods

### 2.1. Participants

Participants were 43 infants with DS. At Time 1, chronological age ranged from 3.9–17.6 months, M = 9.0 months SD = 3.9, and developmental age was approximately 6 months, SD = 2.6, as measured by the Cognitive scale of the Bayley Scales of Infant and Toddler Development, Third Edition (BSID-III) [44]. At Time 2, chronological age ranged from 9.7 to 24.2 months, M = 15.4, SD = 4.0, and the group developmental age was approximately 10 months, SD = 2.4 (see Table 1 for complete descriptions of age and BSID-III Cognitive scores). There were approximately equal numbers of males and females in the study (53.5% male). The majority of participants were White (79%) and not Hispanic (70%). Six additional infants were assessed at Time 1, but were lost to attrition, and therefore are not included in the study because of the need for complete data for subsequent analyses. There were no meaningful differences in participant characteristics between those infants who did and did not return for Time 2 (i.e., developmental or chronological age, prematurity status, presence of CHD, or maternal/paternal education level).

### 2.2. Procedures

Participants were recruited through regional DS associations, clinics, and support groups across the United States and western Canada using mailings, listservs, and social media. Informed consent was obtained for each subject before they participated in the study and the study procedures were approved by the Institutional Review Board at Colorado State University (19-8991H, approved October 2015). Infants and their caregivers participated in two data collection visits that were six months apart. During Time 1, the BSID-III, developmental history, and IBQ-R were administered. At Time 2, the BSID-III and CSBS-ITC were completed. Study visits took place in the infant’s home, DS organization or other child-friendly spaces, or laboratory space at Colorado State University. Visits lasted approximately 90 min. Primarily mothers (95%) completed the study questionnaires about their infant. Data were collected as part of a broader intervention study and a subset of infants participated in a parent-mediated intervention (n = 28) to promote early reaching behavior. Those who received targeted treatment were compared to the group of participants who did not, which consisted of participants in the control group or participants who did not meet the intervention’s study criteria. Intervention condition was controlled for in longitudinal analyses and did not impact outcome variables of interest (see Results).

### 2.3. Measures

#### 2.3.1. The Bayley Scales of Infant and Toddler Development, Third Edition (BSID-III)

The BSID-III is a standardized assessment for children aged 1–42 months old that measures cognition, receptive communication, expressive communication, fine motor skills, and gross motor skills [44]. The BSID-III Cognitive scale demonstrates adequate concurrent validity with the Wechsler Preschool and Primary Scale of Intelligence-Third Edition (0.72–0.79) [44]. A trained graduate student administered the BSID-III. Cognitive raw scores, standard scores, and developmental age equivalents were used for descriptive purposes and Cognitive raw scores were selected to estimate cognitive ability in the regression analysis.

#### 2.3.2. Child Developmental and Family History

Caregivers completed a survey to collect demographic information on race, ethnicity, child diagnosis, prematurity status, presence of CHD, and child gender. Information on maternal and paternal education levels was also collected. Caregivers reported their education level by selecting from one of the following categories: some high school, high school graduate, 1–3 years of college, college graduate, some graduate school or terminal masters, or professional degree. One caregiver completed the survey and reported on both mother and father education information. Prematurity status, CHD presence, and both maternal and paternal education variables were used to examine differences in regulatory function and early social communication.

#### 2.3.3. Regulatory Function

The Infant Behavior Questionnaire-Revised (IBQ-R) Short Form was completed at Time 1 [34]. The IBQ-R is a caregiver-report questionnaire that measures 14 infant characteristics using 91 items. Questions were answered by the infants’ caregivers using a 7-point Likert scale ranging from “never” to “always”. The IBQ-R is normed for infants 3 to 12 months old and the developmental status of each infant in the current study matched this developmental range, with just one infant participating who had a developmental status of 13 months. The questionnaire demonstrates high internal reliability (*α* = 0.71–0.88) [34,45] and internal consistency was high for each dimension examined in the current study (*α* = 0.73–0.84). The Orienting/Regulation dimension of the existing three-factor structure was used to assess regulatory function [19,34]. The Orienting/Regulation dimension is an average raw score that is calculated from the mean scores of the Duration of Orienting (6 items), Cuddliness/Affiliation (6 items), Low Intensity Pleasure (7 items), and Soothability (7 items) scales [34]. Four of the 6 Cuddliness/Affiliation items and 4 of the 7 Soothability items are reverse scored. The IBQ-R factor dimensions have been evaluated in a sample of children with DS and intercorrelations among dimensions do not differ from typically developing infants [46]. Descriptive statistics were reported for the raw scores of the dimensions (Duration of Orienting, Cuddliness/Affiliation, Low Intensity Pleasure, and Soothability) and Orienting/Regulation factor, and the Orienting/Regulation factor raw score was included in the regression analysis.

#### 2.3.4. Early Social Communication

The Communication and Symbolic Behavior Scales Infant Toddler Checklist (CSBS-ITC) is a developmental screening questionnaire designed for children 6 to 24 months old [42]. The 24-item scale assesses seven predictors of language, divided into three domains: Social, Speech, and Symbolic. The Social domain includes emotion and use of eye gaze, communication, and gestures, the Speech domain examines the use of sounds and words, and the Symbolic domain examines understanding words and the use of objects. Standard scores and percentile rank can be calculated for each domain (Social, Speech, and Symbolic). Standard scores range from 3 to 17 for all three domains and percentile rankings of ≤10% are considered to be in the “range of concern” according to published norms [42]. Social, Speech, and Symbolic raw and standard scores demonstrate good test–retest reliability (0.65–0.88), and the CSBS-ITC is appropriate for use in neurogenetic syndromes [47]. In the current study, internal consistency was high for the domains of the CSBS-ITC (*α* = 0.72–0.77). The CSBS-ITC has also been used in ASD work as a predictor of later ASD symptoms [39] and validated as a screener for communication delays and ASD in infants aged 9–24 months [48]. Raw scores, standard scores, and percentile rank were used in the current study for descriptive purposes and raw scores were used in the subsequent regression analysis.

### 2.4. Data Analysis Plan

The first study objective was to describe score distributions of two caregiver report measures assessing infant regulatory function and early social communication to characterize the nature of within-DS heterogeneity. Descriptive statistics (mean, standard deviation, median, minimum, maximum, skewness, and kurtosis) were examined for the Orienting/Regulation dimension of the IBQ-R and component scales that make up the dimension. Skewness between −1 and 1 and kurtosis between −2 and 2 were selected as a priori criteria for variables to be considered normally distributed. Descriptive statistics were also evaluated for the raw scores, standard scores, and percentile rank of the Social, Speech, and Symbolic domains of the CSBS-ITC. Additional score distribution analyses investigated floor effects and percentage of participants in the range of concern (≤10%) on CSBS-ITC domains.

Next, the predictive value of infant regulatory function for later social communication performance was examined using multivariate multiple regression. Regulatory function at Time 1 and cognitive ability at Time 2 were included in the model predicting social communication at Time 2, measured via the raw scores of the Social, Speech, and Symbolic domains of the CSBS-ITC. All assumptions of multivariate multiple regression were met, including the normal distribution of variables, no violation of multicollinearity, linear relationship among variables, and the absence of sizeable outliers. Within-group variability was examined on the IBQ-R and CSBS-ITC to determine the effects of biomedical comorbidities (prematurity and CHD) and caregiver education level. *T*-tests were used to investigate the relationship between biomedical comorbidities (binary variables) and regulatory function or communication, and ANOVA was used for caregiver education level (ordinal variable with 6 categories). Intervention participation (binary variable) and variables with significant within-group variability were controlled for in the regression analysis.

## 3. Results

### 3.1. Performance on Regulatory Function and Early Social Communication

Average scores for the Orienting/Regulation dimension of the IBQ-R and its four subscales at Time 1 are reported in Table 2. The standard deviations of scales were similar, and no floor effects were observed. However, the Duration of Orienting scale had a larger standard deviation relative to other IBQ-R dimensions, suggesting potentially greater within-group variability on this dimension among infants with DS in the present sample. Biomedical comorbidities (prematurity and CHD) and caregiver education level (maternal and paternal) were examined for their association with IBQ-R dimensions. A significant percentage of the infants were diagnosed with CHD (47%) or were born prematurely (37%). The majority of maternal caregivers had at least some college education. Maternal education involved the following distribution: some high school (7%), high school graduate (7%), 1–3 years of college (23%), college graduate (35%), some graduate school or terminal masters (12%), or professional degree (16%). Paternal caregivers showed similar education levels and involved the following distribution: some high school (5%), high school graduate (16%), 1–3 years of college (21%), college graduate (30%), some graduate school or terminal masters (19%), or professional degree (9%). No significant differences were observed on the Orienting/Regulation dimension based on any of the biomedical comorbidity or caregiver education level variables and all effect sizes were small (see Table 3).

Variability was observed for early communication skills, as indicated by the wide range of scores on the CSBS-ITC (see Table 4 for full CSBS-ITC descriptive statistics). The Social, Speech, or Symbolic domains had similar mean standard scores, although the Speech domain had a more restricted range of performance (maximum standard score of 12). The broad range of percentile rankings reported in Table 4 again indicated within-group variability, even after accounting for chronological age. Raw scores were used for further analyses, as skewness was lower for raw scores compared to standard scores on all three CSBS domains. Between 46.5% and 62.8% of infant participants met the “range of concern” for the domains of this assessment (percentile rank ≤ 10%; 48.8% Social domain, 46.5% Speech domain, and 62.8% Symbolic domain). Proportions of participants who scored at the floor on standard scores ranged from 7.0% to 25.6% (11.6% Social domain, 7.0% Speech domain, and 25.6% Symbolic domain) and no raw score floor effects were observed. Within-group variability was also examined for the CSBS-ITC based on biomedical comorbidities (prematurity and CHD) and caregiver education level (maternal and paternal). Variability in scores on the CSBS-ITC domains were observed based on prematurity status and effect sizes were medium to large (see Table 3). The observed effects of prematurity on the CSBS-ITC communication domains justified the inclusion of the variable in the subsequent regression analysis. No significant differences were observed in the CSBS-ITC based on CHD or caregiver education level.

### 3.2. Relationship Between Regulatory Function and Early Social Communication

A multivariate multiple regression was completed to examine the relationships between regulatory function, cognitive ability, and early social communication. Participation in the reaching intervention and prematurity were included in the model to control for any potential unanticipated effects on performance. The independent variables included regulatory function (at Time 1), BSID-III Cognitive raw score (at Time 2), intervention participation (yes/no), and prematurity (yes/no). BSID-III Cognitive raw scores were included in the model rather than chronological age to avoid multicollinearity concerns, as the two variables were correlated, *r* (43) = 0.68, *p* < 0.001. Cognitive performance was selected over chronological age because cognition is a more relevant indicator of developmental status. The dependent variables were the raw scores of the three subdomains of the CSBS-ITC: Social, Speech, and Symbolic. This analysis demonstrated that the set of independent variables was related to the set of dependent variables, *F* (12, 96) = 4.99, *p* < 0.001, and the four independent variables accounted for 72.4% of the variance in the CSBS-ITC communication variables. Post-hoc examination of each dimension revealed that regulatory function was related to the set of CSBS-ITC communication variables, when controlling for cognitive ability, intervention participation, and prematurity, *F* (3, 35) = 4.33, *p* < 0.05, *R*^2^ = 0.27. As expected, cognitive ability was also significantly associated with the set of CSBS-ITC communication variables, when controlling for regulatory function, intervention participation, and prematurity, *F* (3, 35) = 12.70, *p* = < 0.001, *R*^2^ = 0.52. Intervention participation and prematurity were not significantly associated with the set of CSBS-ITC communication variables.

Univariate results demonstrated that regulatory function at Time 1 was related to the Social CSBS-ITC performance at Time 2, *F* (1, 42) = 12.19, *p* < 0.01, but not the Speech CSBS-ITC domain, *F* (1, 42) = 0.32, *p* = 0.58, or Symbolic CSBS-ITC domain, *F* (1, 42) = 1.02, *p* = 0.32. There was also a significant association between cognitive ability at Time 2 and the Social domain of the CSBS-ITC, *F* (1, 42) = 33.77, *p* = <0.001, Speech CSBS-ITC domain, *F* (1, 42) = 9.03, *p* < 0.01, and Symbolic CSBS-ITC domain, *F* (1, 42) = 18.55, *p* < 0.001 (see Table 5).

## 4. Discussion

The purpose of the current study was to characterize infant regulatory function and its association with early social communication in infants with DS. DS is associated with challenges in the acquisition of communication and language, and there is a great deal of heterogeneity in outcomes along these dimensions. In this study, we first examined the score distributions of two caregiver report measures of early social communication and regulatory function and found no problematic floor effects with raw scores, however, some floor effects were observed for CSBS-ITC standard scores. Although the standard scores showed no clear mean profile of strengths or weaknesses in communication skill components, the Symbolic domain of the CSBS-ITC demonstrated the largest floor effects and the largest percentage of participants scoring in the “range of concern”. Potential sources of variability, including prematurity, CHD, and caregiver education level, were evaluated. Prematurity was found to be a potential source of variability in communication outcomes, although it was not a significant covariate in subsequent longitudinal analyses. Next, the relationship between regulatory function and communication scales was investigated, and infant regulatory function at Time 1 was found to predict social communication outcomes at Time 2, six months later. These findings suggest that early risk for more pronounced challenges with communication and language foundations may be detectable as early as infancy in DS.

This study contributes to the mounting evidence for the importance of regulatory function for subsequent social communication during early development. Though regulatory function at Time 1 predicted the CSBS-ITC Social domain at the 6-month follow-up (Time 2), this dimension was not associated with the two other CSBS-ITC domains—Speech and Symbolic performance. Therefore, while regulatory function is not a broad predictor of speech and symbolic skill acquisition at the age investigated in the current study, it accounts for variance in early social functioning in infants with DS, including the components of emotion and eye gaze, communication, and gestures. The specificity of this relationship warrants further examination. It is possible that an infant’s ability to organize responses to their environment (i.e., regulatory function) specifically facilitates the development of social skills by supporting infants’ foundational social abilities, such as maintaining attention and directing eye gaze. In addition, early infant attention skills may provide a critical foundation for more advanced social skills (e.g., response and initiation of joint attention or other social overtures). Specifically, infants with the ability to sustain attention to objects or caregiver activity (components of regulatory function) may build on these skills to develop socially modulated eye gaze for joint attention. The relationship between early regulatory function and subsequent social communication may also be contingent upon modulated responses to social aspects of caregiving. It may be the case that high ratings on the Cuddliness and Soothability scales (components of regulatory function) are more likely to evoke positive and responsive actions from their caregiver when compared to an infant who responds more negatively to comforting from a caregiver. These positive responses to caregiving may elicit more frequent social initiations from caregivers and provide an infant with more opportunities to develop an understanding of the social world, thus contributing to the connection between early regulatory function and social skill acquisition.

Although the positive association between regulatory function and social skills aligns well with the broader literature on the association between EF and communication/language development, an inverse relationship has also been previously reported in specific social communication skill areas. Todd and Dixon (2010) found that typically developing infants who received lower regulatory function ratings (i.e., IBQ-R Orienting/Regulation scores) demonstrated higher levels of response to joint attention. The authors interpreted these findings by suggesting that infants with lower levels of regulatory function relied more on social cueing and infants with greater regulatory function were less socially attuned because they were able to regulate independently [26]. The contrast between this previous report and findings from the present study may be explained by issues unique to phenotypic features associated with DS. Because infants with DS often demonstrate slower processing speed and longer latencies to produce planful actions than typically developing infants [32], it is plausible that infants with relatively stronger regulatory skills in the current study are actually performing at a similar level to the typically developing infants with relatively lower levels of regulatory function in previous studies [26]. The infants with DS in the current study who demonstrate lower levels of regulation may not have reached a cognitive status in which they can rely on social cues and are also not independent with their regulatory function. The positive relationship between regulatory function and social skills observed in the current study highlights the importance of understanding early regulatory function in specific neurogenetic syndromes, such as DS, and raises the possibility that they may differ from patterns observed in typical developing infants.

Prematurity, CHD, and parent education level were examined as potential sources of within-group variability in the sample. Communication ratings differed based on prematurity status; however, prematurity was not a significant covariate in the model of the relationship between regulatory function and communication outcomes. Even so, the identified differences in communication performances based on prematurity status are of clinical relevance, especially considering the medium and large effect sizes of the reported differences. Infants born prematurely demonstrated significantly lower Speech and Symbolic domain scores on the CSBS-ITC and, therefore, should be monitored closely for challenges with expressive and receptive language skill acquisition in the first two years of development.

In addition to its ramifications for communication intervention planning, the observed relationship between regulatory function and subsequent social communication outcomes makes a preliminary, yet critical, contribution to the long-term goal of identifying infant precursors to comorbid ASD in DS. Comorbid ASD has profound consequences for well-being and adaptation for individuals with DS. Previous population-based work has estimated the prevalence of comorbid DS and ASD (DS + ASD) at approximately 18% [49], with other recent estimates ranging as high as 42% [50]. Co-occurring ASD in DS is associated with severely challenging maladaptive outcomes, including self-injury [51] and developmental regression [52]. As such, the presence of comorbid ASD has pronounced implications for outcomes in DS and places an added burden on families to seek ASD-related treatments. Addressing the negative impact of comorbid ASD in DS with early intervention could have a widespread impact on a large subgroup of individuals with DS and their families.

Considering that the relationship between early regulation and communication skill acquisition is of clinical relevance for early intervention planning in DS, findings should be interpreted with caution as the study has several limitations that should be considered. A primary limitation of this study is the modest sample size and narrow chronological age range, which reduced statistical power. There were also six infants who were assessed at the first time point who did not return for Time 2. While it is likely that these infants’ data were missing at random, there is a possibility that their performance would have contributed additional variability to the observations in the current study. In addition, the study focused on within-group variability of infants with DS with no comparison group examined. A developmentally equated comparison group of typically developing infants or a group with other neurogenetic syndromes would make it possible to answer additional questions and make direct comparisons regarding the syndrome-specificity of the observed relationship between regulatory function and communication outcomes.

A lack of normative data for the IBQ-R also limited study conclusions regarding strengths and weaknesses of self-regulatory domains. Some participants were tested outside the chronological age norms on the IBQ-R, even though they were mostly within the normed range in terms of developmental level. It is also important to note that both measures of interest in this study were derived from caregiver report, which may have introduced response bias. Future studies should include laboratory measures to reduce this confounding factor. Additionally, the timespan between visits was relatively short (6 months). Further longitudinal follow-up would allow for a more nuanced understanding of variability in social and communication outcomes in infants and young children with DS, including a potential relationship between regulatory function and speech or symbolic domains. Finally, future work should characterize changes and determinants of change from infancy through preschool to gain insight into the lasting impact of infant regulatory function on longer-term communication and language outcomes as young children with DS enter kindergarten.

## 5. Conclusions

This longitudinal study examined the relationship between early regulatory function and communication and language abilities in infants with DS across two time points. The study findings provide important new information indicating that early regulatory abilities predict social skills during infancy in DS. Interventions targeting caregiver facilitation of emerging regulatory abilities in their infants with DS could positively affect communication and language developmental trajectories for this population.

## Figures and Tables

**Table 1:** Infant BSID-III characteristics at Time 1 and 2, *n* = 43.

	Mean (*SD*)	Min	Max	Median	Skew	Kurtosis
Time 1						
Chronological Age	9.0 (3.9)	3.9	17.6	8.5	0.59	−0.96
BSID-III Cognitive Raw Score	27.0 (7.5)	14	42	26	0.16	−0.98
BSID-III Cognitive Scaled Score	6.7 (2.7)	1	14	7	0.45	−0.57
BSID-III Cognitive DA	6.6 (2.6)	3.3	13	6	0.71	−0.51
Time 2						
Chronological Age	15.4 (4.0)	9.7	24.2	14	0.57	−0.87
BSID-III Cognitive Raw Score	37.5 (5.0)	29	53	37	1.31	2.21
BSID-III Cognitive Scaled Score	5.5 (2.3)	1	10	6	−0.05	−0.69
BSID-III Cognitive DA	10.6 (2.4)	7	18	10	1.45	2.52

BSID-III = Bayley Scales of Infant and Toddler Development, Third Edition; DA = Developmental age.

**Table 2:** Characteristics of regulatory function at Time 1.

	Mean (*SD*)	Min	Max	Median	Skew	Kurtosis
IBQ-R Orienting/Regulation	5.2 (0.7)	3.9	6.9	5.3	0.11	−0.44
Duration of Orienting	3.7 (1.5)	1.0	6.8	3.8	−0.06	−0.72
Cuddliness/Affiliation	5.9 (0.8)	4.3	7.0	6.2	−0.83	−0.45
Low Intensity Pleasure	5.6 (1.0)	3.3	7.0	6.0	−0.86	−0.05
Soothability	5.7 (0.7)	4.4	7.0	5.9	−0.26	−0.65

Note: IBQ-R Orienting/Regulation is an average raw score calculated from mean Duration of Orienting, Cuddliness/Affiliation, Low Intensity Pleasure, and Soothability scores.

**Table 3:** Within-group differences based on biomedical comorbidity and caregiver education level.

	Prematurity			CHD			Maternal Education			Paternal Education		
	*t*	*p*	*d*	*t*	*p*	*d*	*F*	*p*	*η* ^2^	*F*	*p*	*η* ^2^
IBQ-R Orienting/Regulation	0.20	0.84	0.06	0.35	0.73	0.11	2.37	0.06	0.24	1.49	0.22	0.17
Social Composite Raw Score	1.49	0.143	0.49	0.51	0.61	0.16	0.91	0.49	0.11	1.08	0.39	0.13
Speech Composite Raw Score	2.09	0.04 *	0.65	0.76	0.45	0.23	0.28	0.92	0.04	0.48	0.79	0.06
Symbolic Composite Raw Score	2.97	0.005 *	0.89	0.76	0.46	0.22	1.85	0.13	0.20	0.73	0.61	0.09

* *p* < 0.05; *d* = Cohen’s *d*.

**Table 4:** Score distributions of the Communication and Symbolic Behavior Scales Infant Toddler Checklist (CSBS-ITC) at Time 2.

	Mean (*SD*)	Min	Max	Median	Skew	Kurtosis
Social Composite Raw Score	15.0 (5.0)	6	26	15	0.10	−0.77
Social Composite Standard Score	6.9 (2.9)	3	17	7	1.11	2.00
Social Composite % Rank	21.8 (24.1)	1	99	16	1.55	1.87
Speech Composite Raw Score	6.1 (2.7)	1	12	6	0.60	−0.14
Speech Composite Standard Score	6.6 (2.2)	3	12	7	0.33	−0.35
Speech Composite % Rank	18.5 (18.3)	1	75	16	1.43	1.52
Symbolic Composite Raw Score	7.1 (3.4)	2	17	6	1.03	−0.59
Symbolic Composite Standard Score	6.1 (3.2)	3	17	5	1.38	2.11
Symbolic Composite % Rank	17.3 (24.5)	1	99	5	1.99	3.52

**Table 5:** Multivariate multiple regression results predicting CSBS-ITC domains.

	CSBS-ITC: Social			CSBS-ITC: Speech			CSBS-ITC: Symbolic		
	*B*	*SE B*	*η* ^2^	*B*	*SE B*	*η* ^2^	*B*	*SE B*	*η* ^2^
Regulatory function	2.56 **	0.73	0.25	0.29	0.52	0.01	0.58	0.58	0.03
Cognitive ability	0.63 **	0.11	0.48	0.23 **	0.08	0.20	0.37 **	0.09	0.33
Intervention participation	1.40	1.88	0.02	0.88	1.33	0.01	1.81	1.48	0.04
Prematurity	0.68	1.96	0.003	0.91	1.38	0.01	2.86	1.54	0.09

Note: Regulatory function = IBQ-R Orienting/Regulation raw score at Time 1; Cognitive ability = BSID-III Cognitive raw score at Time 2; ** *p* < 0.01.

## Data Availability

The data presented in this study are available on request from the corresponding author.
